# Cholesterol-Rich Microdomains Contribute to PAR1 Signaling in Platelets Despite a Weak Localization of the Receptor in These Microdomains

**DOI:** 10.3390/ijms21218065

**Published:** 2020-10-29

**Authors:** Vahideh Rabani, Jennifer Lagoutte-Renosi, Jennifer Series, Benoit Valot, Jean-Marie Xuereb, Siamak Davani

**Affiliations:** 1 EA 3920 Université Bourgogne Franche-Comté, F-25000 Besancon, France; v.rabani@gmail.com (V.R.); jennifer.lagoutte_renosi@univ-fcomte.fr (J.L.-R.); 2 Laboratoire de Pharmacologie Clinique et Toxicologie-CHU de Besançon, F-25000 Besancon, France; 3 Inserm UMR1048, Institut des Maladies Métaboliques et Cardiovasculaires (I2MC), F-31100 Toulouse, France; series.j@chu-toulouse.fr (J.S.); jean-marie.xuereb@inserm.fr (J.-M.X.); 4 UMR CNRS 6249 Chrono-Environnement, Université Bourgogne Franche-Comté, F-25000 Besancon, France; benoit.valot@univ-fcomte.fr

**Keywords:** PAR1, vorapaxar, platelets, microdomains, TRAP

## Abstract

Platelet protease-activated receptor 1 (PAR1) is a cell surface G-protein-coupled receptor (GPCR) that acts as a thrombin receptor promoting platelet aggregation. Targeting the PAR1 pathway by vorapaxar, a PAR1 antagonist, leads to a reduction in ischemic events in cardiovascular patients with a history of myocardial infarction or with peripheral arterial disease. In platelets, specialized microdomains highly enriched in cholesterol act as modulators of the activity of several GPCRs and play a pivotal role in the signaling pathway. However, their involvement in platelet PAR1 function remains incompletely characterized. In this context, we aimed to investigate whether activation of PAR1 in human platelets requires its localization in the membrane cholesterol-rich microdomains. Using confocal microscopy, biochemical isolation, and proteomics approaches, we found that PAR1 was not localized in cholesterol-rich microdomains in resting platelets, and only a small fraction of the receptor relocated to the microdomains following its activation. Vorapaxar treatment increased the level of PAR1 at the platelet surface, possibly by reducing its endocytosis, while its colocalization with cholesterol-rich microdomains remained weak. Consistent with a cholesterol-dependent activation of Akt and p38 MAP kinase in thrombin receptor-activating peptide (TRAP)-activated platelets, the proteomic data of cholesterol-rich microdomains isolated from TRAP-activated platelets showed the recruitment of proteins contributing to these signaling pathways. In conclusion, contrary to endothelial cells, we found that PAR1 was only weakly present in cholesterol-rich microdomains in human platelets but used these microdomains for efficient activation of downstream signaling pathways following TRAP activation.

## 1. Introduction

Protease-activated receptor 1 (PAR1) is the prototypical member of a family of G-protein-coupled receptors (GPCRs) that mediate cellular responses to thrombin, the strongest human platelet activator, and related proteases [1]. Indeed, thrombin irreversibly activates PAR1 by cleaving the amino-terminal exodomain of the receptor, which exposes a tethered peptide ligand that binds the heptahelical bundle of the receptor to induce G-protein activation via an intramolecular signaling mechanism [2]. PAR1 activation is based on a process named biased signaling, with a ligand-dependent preferential activation of downstream targets [1], via several G proteins, including Gα_q_, Gα_12/13_, and Gα_i_, as well as Gβγ [3].

In humans, most of the studies on PAR1 have been performed in endothelial cells showing that their localization in raft microdomains plays a pivotal role in presenting its exodomain for interaction with coagulation proteases [4]. In all cellular membranes, raft microdomains are mesoscale domains of 2 to 20 nm in diameter, highly enriched in cholesterol, sphingolipids, and proteins [5].

In endothelial cells, agonists’ interaction with PAR1 induces cleavage of PAR1 by either activated protein C (APC) or thrombin in lipid rafts [6], initiating signaling responses in endothelial cells [4]. In addition, it has been shown that in human endothelial cells, PAR1 needs to partition into raft microdomains and cofractionate with caveolin-1, in caveolar raft microdomains, to be activated [7]. Lipid raft microdomains also play a pivotal role in several signaling pathways leading to platelet activation [8]. These cholesterol-rich microdomains are the focus of attention in biological membrane research and are implicated in cardiovascular diseases through their participation in platelet activation [9,10], and in the mechanisms mediating drug effects [11,12].

Microdomains are well known as modulators of the activity of a number of GPCRs [13]; however, their involvement in PAR1 function in platelets remains incompletely characterized. Thus, our aim was to investigate whether PAR1 is located in membrane cholesterol-rich microdomains in human platelets, and whether its activation requires these microdomains. We also tested the effect of vorapaxar, which is a first-in-class PAR1 antagonist approved by the Food and Drug Administration for the reduction of atherothrombotic events in patients with a history of myocardial infarction [14]. Vorapaxar is an orthosteric selective antagonist that binds nearly irreversibly to the ligand binding pocket on the extracellular surface of PAR1 [6]. In endothelial cells, vorapaxar inhibits the vast majority of PAR1 downstream signaling [3,15]. In human platelets, vorapaxar inhibits thrombin-mediated platelet activation [16,17].

## 2. Results

### 2.1. Analysis of the Colocalization of PAR1 and Cholesterol-Rich Microdomains

**Figure 1:**
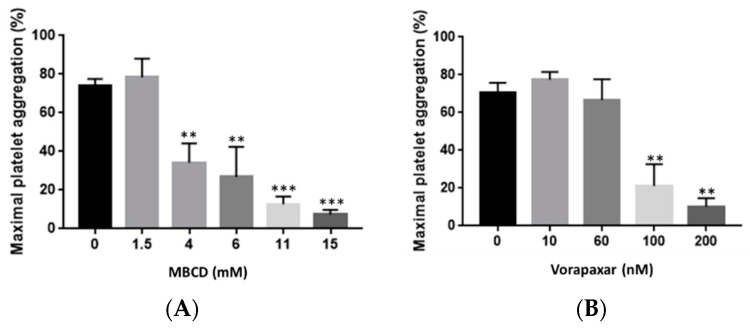
Effect of MBCD and vorapaxar on TRAP-activated washed platelets. (**A**) MBCD significantly decreased the maximal platelet aggregation induced by TRAP 25 µM. Results are mean ± SEM from three independent experiments. ** *p* < 0.01, *** *p* < 0.001 according to one-way ANOVA test. (**B**) Vorapaxar at 100 and 200 nM significantly decreased the maximal platelet aggregation induced by TRAP 25 µM. Results are mean ± SEM from three independent experiments. ** *p* < 0.01 according to one-way ANOVA test. MBCD: methyl β cyclodextrin.

The experimental conditions for platelet stimulation and inhibition were optimized by using light transmission platelet aggregometry assays. Stimulation of washed platelets by 25 µM thrombin receptor-activating peptide (TRAP) for 10 min induced a potent platelet aggregation response, which was inhibited by increasing doses of methyl β cyclodextrin (MBCD) (Figure 1A). Addition of vorapaxar 100 nM strongly inhibited platelet aggregation induced by 25 µM TRAP (Figure 1B).

**Figure 2:**
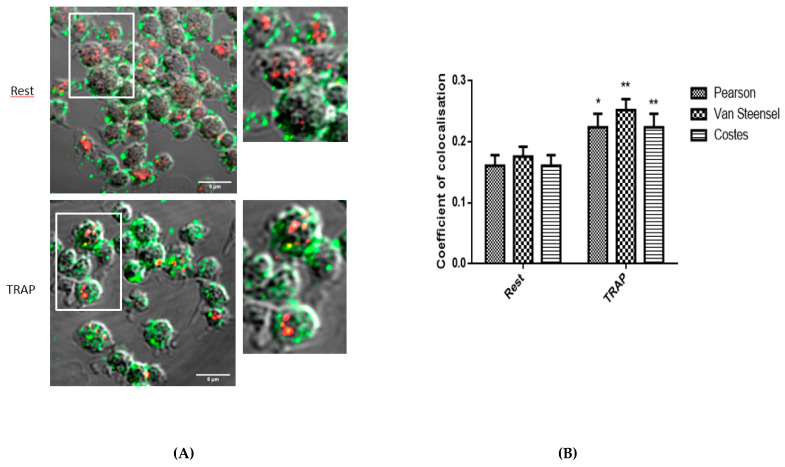
Weak colocalization of PAR1 and cholesterol-rich microdomains in human platelets. (**A**) Double staining of PAR1 (green) and cholesterol-rich microdomains (red) showing no significant colocalization of PAR1 in cholesterol-rich microdomains in resting (Rest) platelets and a weak but significant increase of colocalization in TRAP-stimulated platelets. Images shown are representative of five independent experiments (15 images were analyzed). The insets show an enlargement of the indicated region (scale bar = 5 µm). (**B**) Statistical tests for measures of colocalization between PAR1 and cholesterol-rich microdomains confirm a weak but significant increase in TRAP-activated platelets. This colocalization remained low as the coefficient of colocalization measured by different tests including Pearson’s, Van Steensel’s, and Costes’ was lower than 0.5. * *p* < 0.05, ** *p* < 0.005 (according to a student *t* test), *n* = 5.

To investigate whether PAR1 localized in cholesterol-rich microdomains, we first used a confocal imaging approach. Cholera toxin, a well-known marker of cholesterol-rich microdomains, was used as well as a specific PAR1 antibody able to decorate the receptor at the platelet surface. As shown in Figure 2A, in resting platelets, a very weak colocalization between PAR1 and cholesterol-rich microdomains was observed. The Pearson correlation coefficient, Van Steensel’s cross-correlation coefficient, and Costes’ overlap coefficient used to quantify the degree of colocalization between the two fluorophores, indicated little co-occurrence as they were all below 0.2 (Figure 2B). This colocalization significantly increased following platelet stimulation by TRAP but remained weak (<0.3) (Figure 2A,B). As shown in Supplementary Figure S1A,B, addition of vorapaxar increased the labeling of PAR1 at the surface of TRAP-stimulated platelets, possibly due to blockade of its internalization. The degree of colocalization of PAR1 and cholesterol-rich microdomains remained weak in the presence of vorapaxar.

**Figure 3:**
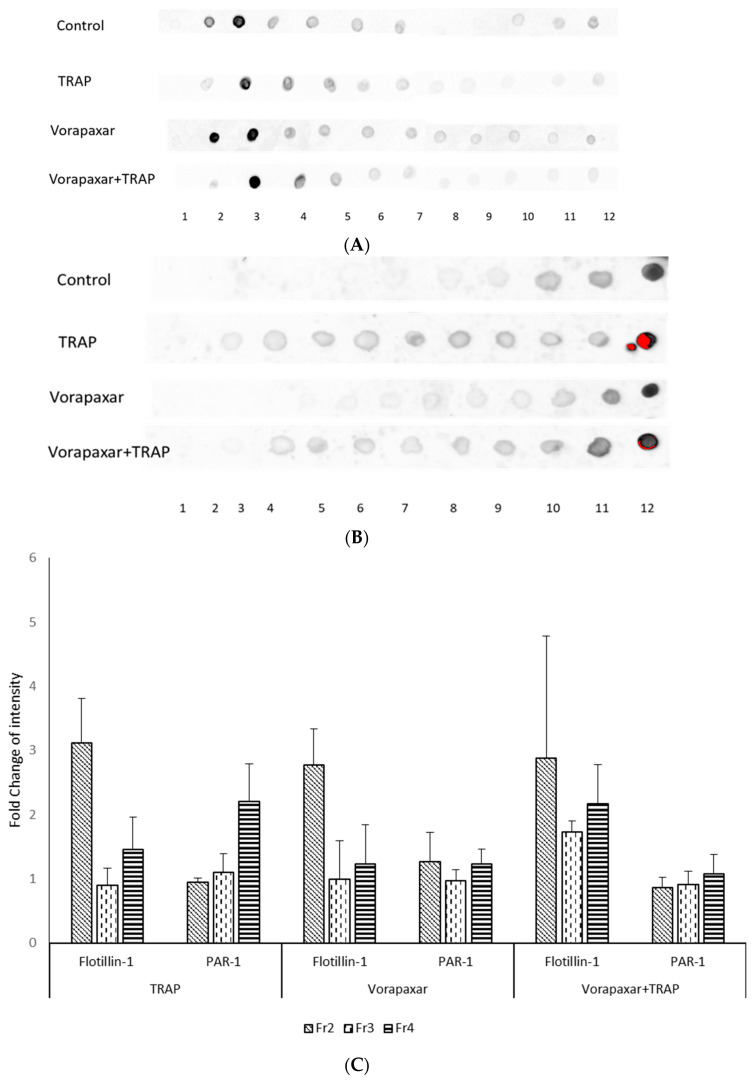
Assessment of colocalization of PAR1 and cholesterol-rich microdomains by a biochemical approach. (**A**) Flotillin-1, a marker of cholesterol–rich microdomains, was mainly detected in fractions 2–4 in all experimental groups; (**B**) PAR1 was mainly present in fractions 10–12, which did not contain cholesterol-rich microdomains; (**C**) Quantitative dot blot of flotillin-1 and PAR1 in experimental groups compared to control.

We then investigated the colocalization of PAR1 and cholesterol-rich microdomains in the different experimental groups by biochemical isolation of cholesterol-rich microdomains and immunoblot experiments. The fractions of the gradient containing cholesterol-rich microdomains were identified based on the presence of flotillin-1, a well-known cholesterol-rich microdomain resident protein. Dot blot analysis showed the presence of flotillin-1 in fractions 2–4 (Figure 3A). Consistent with the imaging experiments, in resting platelets, PAR1 was not detected in the flotillin-1-rich fractions and was mainly present in fractions 10–12 of the gradient (Figure 3B). Following TRAP stimulation, we observed a weak presence of PAR1 in fractions 2–4. This was also found in the vorapaxar + TRAP group (Figure 3B). These results were confirmed by quantification analysis of the dot blots (Figure 3C). Overall, the results obtained by confocal imaging are corroborated by the biochemical approach indicating that PAR1 was not present in cholesterol-rich microdomains in resting human platelets. Following TRAP activation, a small proportion of the receptor was relocated in the microdomains. Moreover, PAR1 was not detected in cholesterol-rich microdomains isolated from different experimental groups by mass spectrometry proteomics analysis.

### 2.2. Impact of Cholesterol Depletion and Vorapaxar on Akt and p38 MAP Kinase Activation in TRAP-Stimulated Platelets

#### 2.2.1. Flow Cytometry Analysis

**Figure 4:**
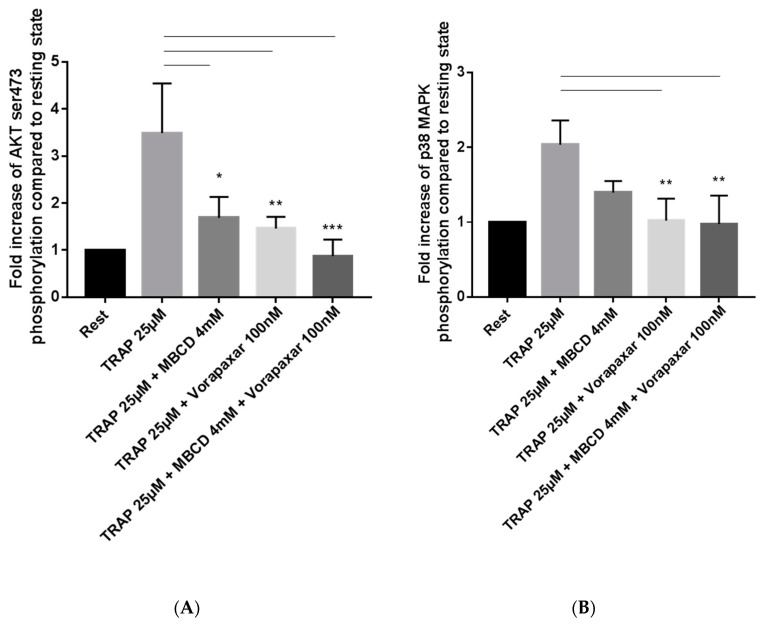
Effect of MBCD and vorapaxar on Akt and p38 MAP kinase phosphorylation following TRAP activation. (**A**) TRAP (25 µM)-induced phosphorylation of Akt on Ser 473 was significantly decreased in MBCD-treated platelets. As expected, vorapaxar (100 nM) strongly impaired the phosphorylation of Akt on Ser 473 following TRAP activation. The association of vorapaxar and MBCD treatment abolished TRAP-induced Akt phosphorylation on Ser473. (**B**) MBCD treatment tended to decrease the phosphorylation of p38 MAP kinase in TRAP (25 µM)-activated platelets. Vorapaxar (100 nM) significantly inhibited TRAP-induced p38 MAP kinase phosphorylation. Results are mean ± SEM from four independent experiments. * *p* < 0.05, ** *p* < 0.01, ***, *p* < 0.001 according to one-way ANOVA. MBCD: methyl β cyclodextrin, TRAP: thrombin receptor-activating peptide.

In platelets, Akt and p38 mitogen-activated protein (MAP) kinase are two important signal transduction pathways downstream of GPRCs. To analyze these pathways, we studied by flow cytometry the level of Akt phosphorylation on Ser473 and the level of p38 MAP kinase phosphorylation on Tyr180/182. As expected, the phosphorylation of Akt and p38 MAP kinase strongly increased in platelets activated by TRAP 25 µM. Treatment with MBCD significantly decreased TRAP-induced Akt phosphorylation and tended to decrease p38 MAP kinase phosphorylation (Figure 4A,B and Supplementary Figure S2). Addition of vorapaxar inhibited TRAP-induced Akt phosphorylation and prevented the phosphorylation of p38 MAP kinase (Figure 4A,B). These results suggest that in TRAP-activated platelets, Akt and p38 MAP kinase signaling requires membrane cholesterol and likely membrane integrity.

#### 2.2.2. Proteomic Analysis

**Figure 5:**
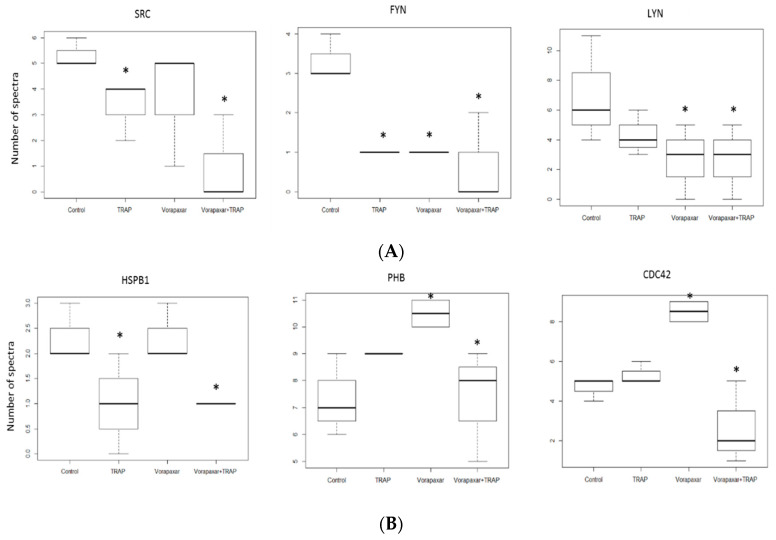
Proteomic analysis of Akt and MAP kinase signaling pathways. (**A**) Akt proteomic analysis showed significant differences in expression level of SRC, FYN, and LYN. * *p* < 0.05 according to one-way ANOVA test. (**B**) Proteins participating in MAP kinase pathway which showed significant difference between different experimental groups. * *p* < 0.05 according to one-way ANOVA test. Number of spectra: visualized spectra used to identify peptides and proteins by the X!Tandem search engine. Src: proto-oncogene tyrosine-protein kinase; HSPB1: heat shock protein beta-1; PHB: prohibitin; CDC42: cell division control protein 42.

Proteomes of cholesterol-rich microdomains isolated from the different groups focusing on MAP kinase, Akt, and “thrombin signaling through proteinase-activated receptors” signaling pathway were cross-referenced with the participating proteins of these signaling pathways via the REACTOM database, in order to investigate the relation between these signaling pathways and cholesterol-rich microdomains. From 275 proteins participating in Akt signaling (ID: R-HSA-1257604.8), 12 proteins were found in the proteomes of isolated microdomains. Among these, several proteins showed a significant difference between experimental groups (Figure 5A).

Our results showed that expression of Src proto-oncogene tyrosine-protein kinase decreased significantly in TRAP and vorapaxar + TRAP groups compared to the control (*p* < 0.05) while, in the vorapaxar group, a nonsignificant difference was found compared to the control group. Tyrosine-protein kinase Fyn showed a significantly lower level in all groups compared to the control (*p* < 0.05). Considering the starter role of Lyn and Fyn in cascade activation of platelets [18], we analyzed levels of Lyn also, although it is not in the Akt signaling pathway. Lyn showed a significantly lower number of spectra in vorapaxar and vorapaxar + TRAP platelets compared to controls (*p* < 0.05), but not between TRAP and control groups (Figure 5A).

From 305 proteins participating in the MAP kinase signaling pathway (ID: R-HSA-5683057.3), we found 26 proteins in proteomes of isolated microdomains. Among these proteins, we focused on three that showed significant differences between the different experimental groups. Proteins from this signaling pathway showed a higher presence in cholesterol-rich microdomains than proteins from the Akt signaling pathway (26 vs. 12 proteins). Our results show that heat shock protein beta-1 (HSPB1) decreased in TRAP and vorapaxar + TRAP compared to control (*p* < 0.05) (Figure 5B). These results suggest that treatment by vorapaxar did not affect its expression in cholesterol-rich microdomains, contrary to the TRAP group. Prohibitin (PHB), a protein associated with PAR1 activation, showed a higher level in TRAP, vorapaxar, and vorapaxar + TRAP groups compared to the control group (*p* < 0.05) (Figure 5B). In addition, we observed higher expression of PHB in the vorapaxar group compared to other groups. Cell division control protein 42 (CDC42) showed higher levels in the vorapaxar group and significantly lower levels in the vorapaxar + TRAP group (*p* < 0.05) (Figure 5B).

**Figure 6:**
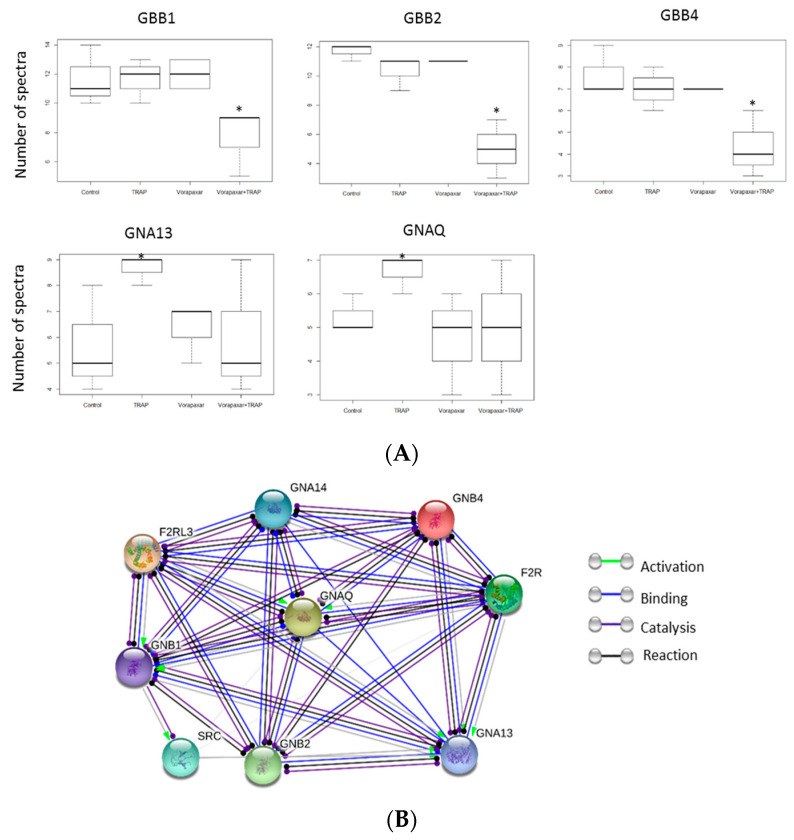
Thrombin signaling through proteinase-activated receptor participating proteins. (**A**) Proteins which showed significant difference in number of spectra in experimental groups. * *p* < 0.05 according to one-way ANOVA test. (**B**) Network analysis of proteins detected in thrombin signaling through proteinase-activated receptors. GBB: guanine nucleotide-binding protein GI/GS/GT subunit beta; GNA: guanine nucleotide-binding protein subunit alpha; GNAQ: guanine nucleotide-binding protein G(q) subunit alpha; GNB: guanine nucleotide-binding protein subunit beta; Src: proto-oncogene tyrosine-protein kinase; PAR: protease activated receptor; F2R (PAR1): coagulation factor II thrombin receptor; F2RL3 (PAR4): F2R like trypsin receptor 3.

In same manner, we studied the “thrombin signaling through proteinase-activated receptors (PARs)” (ID: R-HSA-456926.1) signaling pathway. Among 31 proteins participating in this pathway, 8 were observed in the cholesterol-rich microdomains of experimental groups presented in network analysis (Figure 6A,B). Guanine nucleotide-binding protein G(I)/G(S)/G(T) subunit beta-1 (GBB1), beta-2 (GBB2), and beta-4 (GBB4) showed a significant decrease in the vorapaxar + TRAP group compared to other groups (*p* < 0.05). Guanine nucleotide-binding protein subunit alpha-13 (GNA13) and guanine nucleotide-binding protein G (q) subunit alpha (GNAQ) showed a significant increase in TRAP compared to all other groups (*p* < 0.05). There was no significant difference in GNA14 (guanine nucleotide-binding protein subunit alpha-14) and PAR-4. Src, which participates in this pathway, was previously studied in the Akt signaling pathway.

## 3. Discussion

To the best of our knowledge, this is the first study to analyze the colocalization of PAR1 and cholesterol-rich microdomains in platelets. Our major findings are as follows: (i) in resting human platelets, PAR1 very weakly colocalized with cholesterol-rich microdomains; (ii) TRAP stimulation increased the colocalization of PAR1 and cholesterol-rich microdomains, which remained, however, weak; (iii) cholesterol depletion affected Akt and p38 MAP kinase activation downstream of TRAP stimulation; (iv) cholesterol-rich microdomains contained proteins essential to PAR1 signaling pathway in platelets activated by TRAP.

In 1997, Simons et al. proposed the concept of specialized microdomains of the plasma membrane. This concept, named lipid raft theory, was based on the presence of organized microdomains enriched in glycophospholipids, cholesterol, and proteins [19]. These microdomains can be isolated in detergent-resistant membrane (DRM) form by nonionic detergent from other soluble parts of the membrane [20]. These cholesterol-rich microdomains act as platforms for the assembly of membrane receptors and are involved in signal transduction cascades, ion channel function, and apoptosis [21,22].

PARs have multiple important functions in protease signaling in cells. There are four members of this family of G-protein-coupled receptors (PAR1–4). In this paper, we aimed to investigate whether activation of PAR1 in human platelets requires its localization in the membrane cholesterol-rich microdomain.

Indeed, we specifically focused on PAR1 in human platelets because PAR3 is expressed at very low levels in humans, although it is highly expressed on mouse platelets. Concerning PAR4, its expression is more important in human platelets than PAR3 [23]. Interestingly, PAR3 and PAR4 have an emerging role as an allosteric regulator of PAR1 on endothelial cells or mouse platelets, respectively, by forming heterodimers [24]. Thus, PAR1 serves as cofactor for thrombin-mediated PAR4 activation [23]. In a previous study, PAR4 was not found in cholesterol-rich microdomains in human platelets [25]. Thus, in this study, we investigated whether PAR1 needs to be located on cholesterol-rich microdomains for its function.

Imaging and biochemical approaches indicated that in resting platelets, the colocalization of PAR1 and cholesterol-rich microdomains was very low and weakly increased following TRAP stimulation. Contrary to endothelial cells [7], our results suggest that in platelets activated by TRAP, PAR1 does not need to be localized on cholesterol-rich microdomains. Despite a weak presence of PAR1 in cholesterol-rich microdomains, cholesterol depletion by MBCD leading to disorganization of the membrane and microdomains. Cholesterol depletion decreased significantly TRAP-induced platelet aggregation, TRAP-induced Akt activation and TRAP-induced p38 MAP kinase activation. Akt plays a crucial role in platelet activation-induced signaling of GPCRs [26]. Moreover, it has been shown that p38 MAP kinase is implicated in TRAP-stimulated platelets [27].

Proteomics analysis is an ideal tool to investigate in vitro changes in platelets because of their limited protein synthesis capacity, due to the absence of nucleus and their rather stable proteome [28]. Consistent with the imaging and biochemical data, our proteomic analysis failed to detect PAR1 in isolated cholesterol-rich microdomains, suggesting again its negligible presence in these microdomains.

Our results show that the expression of Src, a tyrosine kinase involved in Akt and MAP kinase signaling pathways, decreased significantly in cholesterol-rich microdomains isolated from TRAP and vorapaxar + TRAP groups. Src is the most abundant Src family of kinases in human platelets and is essential for initiating and propagating signals from αIIbβ3 [29]. Therefore, a decrease in this protein in two experimental groups activated by TRAP could illustrate a relation between thrombin activation and integrin signal propagation [30]. Séverin et al. [31] showed that Fyn and Lyn can be localized in cholesterol-rich microdomains in platelets, whereas Src is excluded from these domains. Maybe Src exclusion is not important, as it has been claimed that Src family kinases play a minor role in aggregation and secretion to low concentrations of *G*_i_ and *G*_q_ protein-coupled receptor agonists [31]. Indeed, these three key tyrosine kinases in platelets showed different trends in TRAP activation or vorapaxar treatment in human platelet cholesterol-rich microdomains.

CDC42 is a key regulator in the dynamics of the actin cytoskeleton and budding in activated platelets [32]. Therefore, this increase in the vorapaxar group calls for more investigation of the relation between vorapaxar and the membrane, and whether the effect of fixing proteins on the membrane, as we observed with PAR1, can be reproduced with other proteins or not.

It has been shown that heat shock proteins can participate in signaling scaffolds, helping to regulate function, including platelet adhesion and spreading via modulation of protein phosphatase activity, and are also involved in controlling actin polymerization during the platelet shape change and subsequent aggregation [33]. It has been shown also in endothelial cells that heat shock proteins are associated with rafts to facilitate integrin activation [34]. Decreases in levels of this protein after TRAP activation on rafts of platelets is not in accordance with these studies.

Prohibitin exhibits a propensity to oligomerize with enrichment in cholesterol-rich microdomains and it is associated with PAR1 activation [35]. Our data showed the highest level of PHB in the platelets treated by vorapaxar, which is in accordance with our confocal results, showing that vorapaxar can have a weak impact on PAR1 localization in cholesterol-rich microdomains.

We showed that Akt phosphorylation is stronger through thrombin activation, but our proteomics analysis showed that MAP kinase shows more changes in the number and level of expression of its contributing proteins in different experimental groups. Therefore, MAP kinase and Akt pathways carry the same value in signal transduction through cholesterol-rich microdomains of platelets in activation or inhibition of PAR1, one with a higher level of phosphorylation of its key player, and the other by keeping a greater number of participating proteins.

It has been shown that Akt phosphorylation for platelet activation can be stimulated by supplemental Gi or Gz signaling [36], and Gα_q_-deficient (GNAQ) mice failed to trigger Akt phosphorylation by thrombin activation [36]. These results suggest that GNAQ plays a role in triggering Akt phosphorylation through thrombin activation. Here, our results showed that GNAQ increased after TRAP activation and decreased after vorapaxar treatment compared to the TRAP group in cholesterol-rich microdomains. This result could suggest that vorapaxar may have an indirect downregulation impact on Akt phosphorylation and thereby on platelet activation.

Network analysis of these proteins related to PAR1 showed that GNA13 was activated by PAR1, PAR4, and GBB2. These results express schematically the finding of Leger et al., who demonstrated a link between PAR1 and PAR4 [37]. GBB2 and PAR4 did not show differences after activation or vorapaxar treatment. We did not observe PAR1 in cholesterol-rich microdomains of experimental group proteomes, but confocal and immunoblot studies showed a weak but higher level of PAR1 in cholesterol-rich microdomains after activation. Therefore, GNA13 could reflect the changes of PAR1 in cholesterol-rich microdomains. This hypothesis is in line with our findings on the effect of vorapaxar in accumulation of PAR1 on the membrane, as the level of GNA13 increases, but not significantly, in the presence of vorapaxar, and we found more PAR1 on the membrane by microscopy.

Furthermore, it has been claimed that orthosteric PAR1 antagonists such as vorapaxar inhibit all signaling downstream of PAR1 and exposure of endothelial cells to nanomolar concentrations of vorapaxar induces endothelial cell barrier dysfunction and apoptosis [15]. Considering our results, we may hypothesize that the effect of vorapaxar on PAR1 downstream signaling is not the same in platelets and endothelial cells, which consequently complicates our understanding of PAR1 mechanisms in platelets. Given the different roles of PAR1 in platelets and endothelial cells, this may explain the different localization of this receptor. Indeed, in endothelial cells, stimulation of PAR1 by thrombin results in the activation of signaling effectors that promote permeability of the endothelial barrier, whereas in platelets, PAR1 activation leads to platelet activation [38]. Overall, our results suggest that the PAR1 signaling pathway is different in platelets from its mechanism in endothelial cells.

## 4. Materials and Methods

### 4.1. Experimental Groups

To investigate the role of platelet cholesterol-rich microdomains on signaling pathways initiated by PAR1, we defined four experimental groups: (i) control group; (ii) platelets stimulated by thrombin receptor-activating peptide (TRAP) (S7152, Sigma-Aldrich, Saint Louis, MI, USA); (iii) platelets treated by vorapaxar (ALSACHIM, Illkirch, France); (iv) platelets treated by vorapaxar and then stimulated by TRAP (hereafter vorapaxar + TRAP). Methyl β cyclodextrin (MBCD) was used to deplete cholesterol in platelet membrane in the different groups. Concentrations of all reagents were determined by aggregometry analysis.

### 4.2. Platelet Preparation

Platelets were obtained from healthy donors from the French Blood Transfusion Center (Etablissement Français du Sang Bourgogne—Franche-Comté—Besançon, France). According to the agreement between the EFS and the Bourgogne Franche-Comté University (convention ref. DECO-15-0178), EFS delivered anonymized samples after blood donors (healthy adults) gave written informed consent that specified the exclusive research purpose and the respect of ethical guidelines. Platelets were received in the laboratory for experiments on the same day as collection. To prepare washed platelets, platelet concentrations were centrifuged after adding 0.5 µL/mL of prostacyclin (PGI2) to separate platelet-poor plasma. Platelet-rich plasma (PRP) was resuspended in adapted HEPES buffer A (140 mM NaCl, 5 mM KCl, 5 mM KH_2_PO_4_, 1 mM MgS0_4_ + 7H_2_O, 10 mM HEPES free acid, bovine serum albumin (BSA), glucose, and PGI2). After centrifugation, platelets were resuspended in HEPES buffer B containing Ca^2+^ 1M without BSA, containing apyrase to obtain 2x10^8^ pl/mL, incubated for 45 min.

### 4.3. Platelet Aggregation

According to experimental groups, washed platelets were incubated with vehicle, MBCD (0, 1.5, 4, 6, 11, and 15 mM) for 10 min, vorapaxar (0, 10, 60, 100, and 200 nM) for 10 min, or TRAP (25 µM) for 10 min or both vorapaxar + TRAP. Platelet aggregation experiments were monitored under continuous stirring at 900 rev min^−1^ at 37 °C using a turbidimetric method and ATP secretion was recorded by measuring the luminescence from the firefly luciferin–luciferase reaction using the Chrono-log^®^ aggregometer (Kordia, Leiden, The Netherlands).

### 4.4. Analysis of Platelet Signaling Pathways by Flow Cytometry

Washed platelets (4 × 10^8^/mL) treated or not by MBCD (4 mM for 10 min) were stimulated by TRAP 25 µM for 10 min and fixed in Cellfix (final concentration 3% adding to distilled water) for 15 min at 37 °C. After permeabilization for 15 min with 0.4% Triton X-100 in PBS at room temperature, platelets were incubated in ice-cold methanol overnight at −20 °C. Samples were then washed in phosphate-buffered saline (PBS) to eliminate methanol and platelets were incubated for 1 h at room temperature with primary phospho-specific antibodies (Akt (ser473), p38MAPK (tyr180/182)) diluted at 1:100 in Tris-buffered saline (TBS) containing 3% bovine serum albumin (BSA) and 0.4% Triton X-100. A secondary antibody, conjugated to Alexa Fluor 647, was then added to the samples at 1:1000 dilution. After incubation at room temperature for 30 min and sample washing, the analysis was performed using a BD LSRFortessa™ flow cytometer (BD Biosciences, San Jose, CA, USA).

### 4.5. Confocal Microscopy Analysis

After stimulation or not by TRAP (25 µM) for 10 min at 37 °C, washed platelets were labeled by cholera toxin/Alexa594 (10 µg/mL) for 15 min at 4 °C. Platelets were washed in PBS and then stained with mouse anti-PAR1 Fab’ (1/50) and incubated overnight at 4 °C. Platelets were then washed in PBS and incubated for 30 min with a secondary anti-mouse ^488^Alexa-coupled antibody. After a washing step and cytospin, samples were visualized with an LSM780 confocal microscope operated with Zen software using a 63 × 1.4 numerical aperture Plan Apochromatic objective lens (Carl Zeiss). Colocalization measurement of PAR1 with cholesterol-rich microdomains was performed by JACoP (http://rsb.info.nih.gov/ij/plugins/track/jacop.html) including statistics analysis performed by ICCB (intensity correlation coefficient-based) tools [39].

### 4.6. Isolation of Membrane Cholesterol-Rich Microdomains

Washed and treated platelets were homogenized by Dounce homogenizer on ice in MBS buffer (25 mM MES, 150 mM NaCl, pH 6.5) containing Triton ×100 1% (*w*/*v*) and antiprotease and EDTA solutions (Halt protease inhibitor cocktail Thermo^®^). Lysate in a 45% sucrose solution was overlaid with a sucrose gradient (5% to 35%) in 8 mL of MBS buffer and was centrifuged for 20 h at 34,000 RPM at 4 °C in Sorvall WX with a swinging rotor TH-641 (Thermo Scientific France, Villebon sur Yvette, France). Twelve sucrose fractions (fractions 1 to 12) were collected from the top to bottom of the tube at the end of ultracentrifugation and stored at −80 °C for further analysis. 

Cholesterol-rich microdomain identification was performed as described previously according to expression of flotillin-1 [40].

### 4.7. Immunoblot Analysis

Presence of flotillin-1, a marker of cholesterol-rich microdomains, and PAR1 was evaluated by immunoblot assay on fractionated membrane. Mouse monoclonal anti-PAR1 (61152, BD Biosciences, San Jose, CA, USA) and monoclonal anti-flotillin-1 (610821, BD Biosciences, San Jose, CA, USA) were used for immunoblotting analysis. Protein levels in each fraction were quantified by BCA Protein Assay (Thermo Scientific France, Villebon sur Yvette, France). For dot blot analysis, 3 µL of each sample, containing the same quantity of proteins, were put on nitrocellulose membrane directly. 

After blocking nonspecific binding sites for 1 h with BSA 5% in TBS-Tween (TBS with 0.1% Tween 20), membranes were incubated by primary antibodies, horseradish peroxidase conjugated goat anti-mouse IgG H & L (a554002, BD Biosciences, San Jose, CA, USA) were used as secondary antibodies.

Dot blot was quantified by ChemiDoc™ XRS+ version 5.1. Quantification of dots was done in a unique area size of membrane since each dot contained the same volume of samples and proteins. The signal intensity of each dot was presented as mean value and calculated by: average intensity of the pixels in defined area subtracted from intensity volume of background. Each membrane of dot blot contained a line of control to validate and normalize reaction with HRP-secondary antibody. Results are presented as mean ± standard deviation (SD) of fold change in intensity of three experiments.

### 4.8. Proteomics Analysis

Based on cholesterol quantification, flotillin-1 positivity, and sucrose density localization, fractions 2–4 in each experimental group were identified as cholesterol-rich microdomains and then were pooled. Proteins of these fractions were precipitated by deoxycholate and trichloroacetic acid (TCA) method and analyzed by mass spectrometry to obtain the proteome of cholesterol-rich microdomain fractions in all experimental groups. This procedure was repeated three times for each experimental condition. The same quantity (25 µL) of each cholesterol-rich microdomain fraction of each experimental group was loaded to 3 SDS-Page gel colored by Colloidal blue. Gels were cut and resuspended in ZUT buffer (Urea 6M, Thirourea 2M, DTT, 10 mM, TrisHcl 30 mM). After second quantification of proteins, samples were subjected to digestion step by trypsin overnight. Digested samples were analyzed by NanoLC-Ultra (Eksigent, London, UK), Version soft: Eksigent 4.1 and Qexactive (Thermo Scientific, Waltham, MA, USA). Peptides were identified based on their spectra, and by using X!Tandem Alanine (2017.2.1.4) software, then protein profiles were constructed. Protein identification was done by coverage of minimum two peptides. Serum albumin and keratins were eliminated during treatments.

In X!Tandem Pipeline, protein identification is performed by the X!Tandem search engine based on visualized spectra used to identify peptides and proteins. Spectra numbers make it possible to perform quantitative comparisons between samples, based on spectral or peptide counting [41].

Post-analysis of proteomics data was performed using different databases such as: Gene Ontology (GO), the Universal Protein Resource (UniProt) retrieval system (http://www.uniprot.org/) [42], STRING [43,44], Venny 2.0 (https://bioinfogp.cnb.csic.es/tools/venny/index2.0.2.html [45], REACTOM [46], and TMHMM 2.0 [47,48], to potential transmembrane domain (TMDs) prediction.

Significant differences in level of spectra of proteins in experimental groups were analyzed by R (http://www.R-project.org) [49].

### 4.9. Statistical Analysis

Data are expressed as mean ± SEM. Significant differences among experimental groups were determined by one-way ANOVA test. Before performing ANOVA, the coefficient of colocalization was determined, and normality of data was tested by the Shapiro–Wilk test. The coefficient of colocalization was determined by Pearson’s, Van Steensel’s, and Costes’ tests. A *p*-value < 0.05 was considered significant. All tests were performed using SigmaPlot software version 12 (Systat Software Inc., San Jose, CA, USA).

## 5. Conclusions

Here, we studied the impact of vorapaxar and TRAP activation on the proteome of cholesterol-rich microdomains of human platelets. We had hypothesized that PAR1 needed cholesterol-rich microdomain localization for activation, as was previously shown to be the case in endothelial cells. Our study shows that contrary to endothelial cells, PAR1 was only weakly present in cholesterol-rich microdomains in human platelets but used them for its function following TRAP activation. However, our proteomics analysis and downstream pathway studies showed that although PAR1 did not require cholesterol-rich microdomain localization to function, it nevertheless used cholesterol-rich microdomains for its signaling pathways. This suggests that we need to elucidate mechanisms of PAR1 in platelets with a view to producing antiplatelet drugs, and not based on its mechanism in endothelial cells. We have shown that activation of PAR1 by thrombin or its antagonism by vorapaxar alters proteomes of cholesterol-rich microdomains. We observed that vorapaxar induced relocalization of sets of proteins in cholesterol-rich microdomain fractions. Study of these modifications suggests that there is some mechanism of effect by vorapaxar on platelets which needs to be elucidated by biological and biochemical studies.

## Supplementary Materials

Supplementary materials can be found at https://www.mdpi.com/1422-0067/21/21/8065/s1.

## Abbreviations

MBCD	Methyl β cyclodextrin
TRAP	Thrombin receptor-activating peptide
PAR1	Protease-activated receptor 1
GPCRs	G-protein coupled receptors
