# Supplementary material for: Cholesterol-Rich Microdomains Contribute to PAR1 Signaling in Platelets Despite a Weak Localization of the Receptor in These Microdomains
Source: Int J Mol Sci. 2020 Oct 29;21(21):8065. doi: 10.3390/ijms21218065 (PMC7663584; doi:10.3390/ijms21218065)
Supplement: Supplementary file 1 [file ijms-21-08065-s001.pdf]

International Journal of
Molecular Sciences

MDPI

Supplementary figure

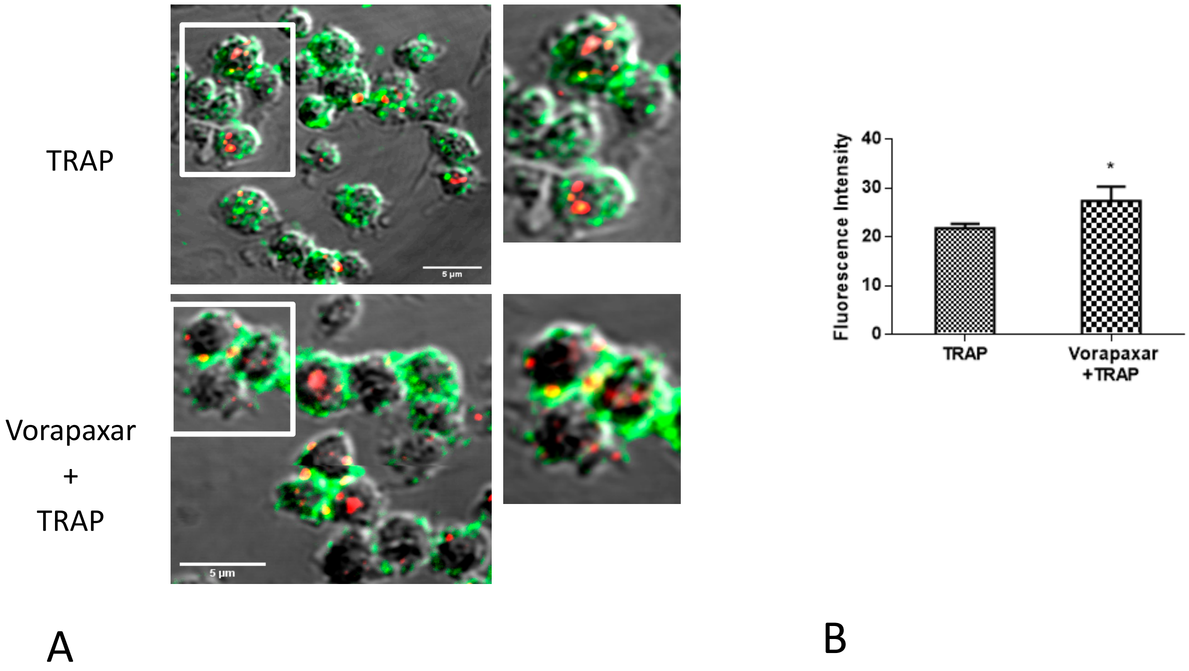

Figure S1: Vorapaxar treatment increases the surface level of PAR1 in TRAP-stimulated platelets (25 µM for 10 min). A) Double staining of PAR1 (green) and cholesterol-rich microdomains (red). The images are representative of 5 independent experiments showing a significant increase in PAR1 staining at the platelet surface in the presence of vorapaxar (100 nM for 10 min). The insets show an enlargement of the indicated region. B) The quantification of the fluorescence intensity (>100 platelets analyzed) confirms the significant increase in PAR1 staining on the platelet surface following vorapaxar treatment. * $p<0.05$, $n=5$.

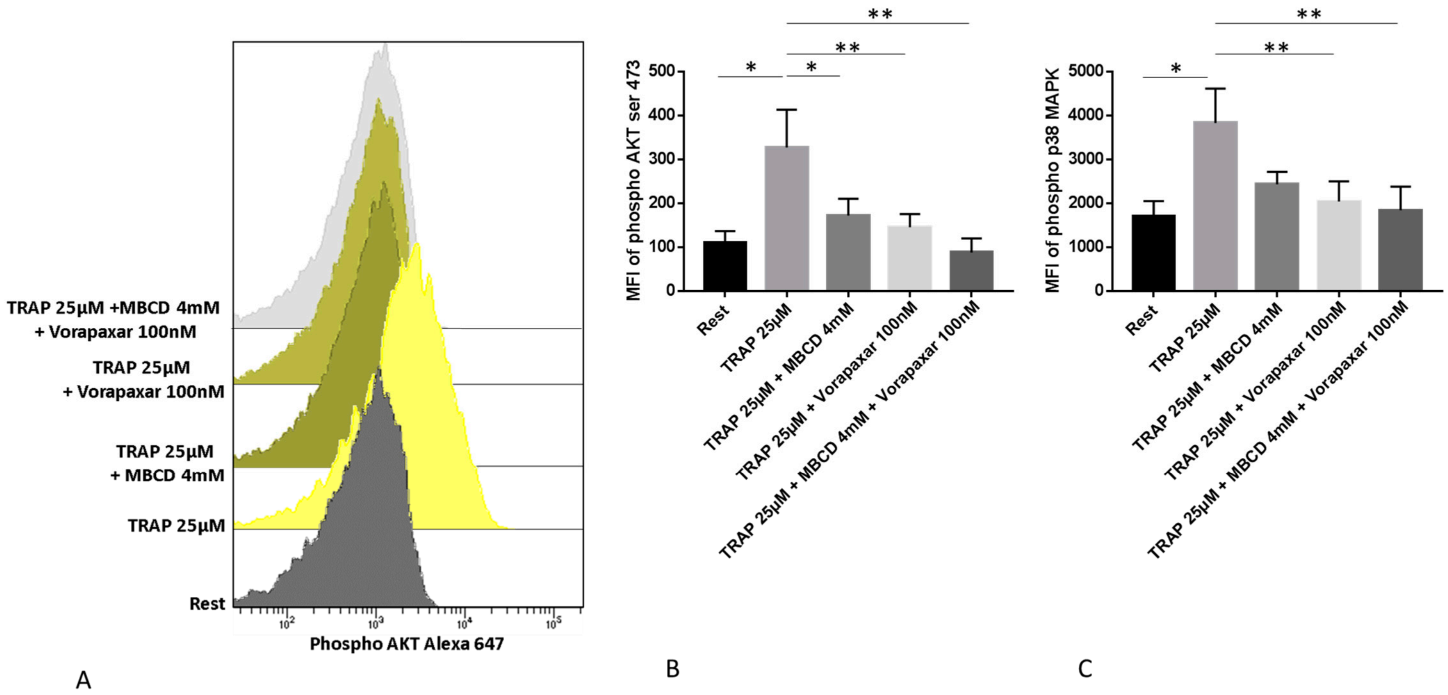

Figure S2: Effect of MBCD and/or vorapaxar on Akt and p38 MAP-Kinase phosphorylation following TRAP activation of human platelets assessed by flow cytometry. (A) A representative image showing the shift in fluorescence intensity illustrating the effect of MBCD and/or vorapaxar on TRAP (25 µM)-induced phosphorylation of Akt on Ser 473. The effect of MBCD (4mM) and/or vorapaxar (100 nM) on the phosphorylation of Akt on Ser 473 (B) and the phosphorylation of p38 MAP-kinase (C) following TRAP activation was quantified by measuring the mean fluorescence intensity (MFI). Results are mean ± SEM from 4 independent experiments. * p<0.05, ** p<0.01, ***, p<0.001 according to One-way ANOVA test.
